# Melatonin Inhibits EMT in Bladder Cancer by Targeting Autophagy

**DOI:** 10.3390/molecules27248649

**Published:** 2022-12-07

**Authors:** Sheng-Yen Hsiao, Chih-Hsin Tang, Po-Chun Chen, Tien-Huang Lin, Chia-Chia Chao

**Affiliations:** 1 Division of Hematology-Oncology, Department of Internal Medicine, Chi Mei Medical Center, Liouying, Tainan City 71004, Taiwan; 2 Institute of Clinical Medicine, College of Medicine, National Cheng Kung University, Tainan City 70101, Taiwan; 3 International Master Program of Biomedical Sciences, China Medical University, Taichung City 40402, Taiwan; 4 Graduate Institute of Biomedical Sciences, China Medical University, Taichung City 40402, Taiwan; 5 Department of Pharmacology, School of Medicine, China Medical University, Taichung City 40402, Taiwan; 6 Chinese Medicine Research Center, China Medical University, Taichung City 40402, Taiwan; 7 Department of Biotechnology, College of Health Science, Asia University, Taichung City 41354, Taiwan; 8 School of Life Science, National Taiwan Normal University, Taipei 10610, Taiwan; 9 Translational Medicine Center, Shin-Kong Wu Ho-Su Memorial Hospital, Taipei 111045, Taiwan; 10 Department of Medical Research, China Medical University Hospital, China Medical University, Taichung 40402, Taiwan; 11 Department of Urology, Buddhist Tzu Chi General Hospital Taichung Branch, Taichung 427213, Taiwan; 12 School of Post-Baccalaureate Chinese Medicine, Tzu Chi University, Hualien 97004, Taiwan; 13 Department of Respiratory Therapy, Fu-Jen Catholic University, New Taipei City 24205, Taiwan

**Keywords:** melatonin, EMT, autophagy, bladder cancer

## Abstract

Melatonin, a naturally biosynthesized molecule secreted by the pineal gland, exhibits antitumor activities against several different types of cancer. The mechanisms of action of melatonin against tumor progression involve cellular apoptosis, antimetastatic activity, antioxidant and mutagenic effects, antiangiogenic activity, and the restoration of cancer immune surveillance. Melatonin has anticancer activity when administered alone or in combination with standard chemotherapeutic agents, with measurable improvements seen in the clinical endpoints of tumor regression and patient survival. However, scant clinical evidence supports the use of melatonin in bladder cancer treatment. Our study has found that melatonin treatment suppresses the bladder cancer cell migratory ability by inhibiting the epithelial-mesenchymal transition (EMT) process, which appears to be linked to melatonin-induced decreases in bladder cancer cell autophagy. Finally, an evaluation of in vivo melatonin-induced antitumor effects in an orthotopic animal model of bladder cancer indicated that melatonin treatment slightly prolonged the survival of tumor-bearing mice. Our study offers novel insights into the use of melatonin in bladder cancer treatment.

## 1. Introduction

Bladder cancer (BCa) is the most common malignancy of the urinary tract and is currently the tenth most common cancer among Taiwanese males [1]. First-line treatment regimens for BCa consist of a transurethral resection of the bladder tumor (TURBT), followed by intravesical instillation of chemotherapeutic agents such as mitomycin, epirubicin and doxorubicin, as well as adjuvant immunotherapy with bacillus Calmette-Guérin (BCG) [2]. However, BCa recurrence is frequent with first-line treatment and often progresses to invasive disease [3], a phenomenon which accounts for the majority of BCa-related deaths. Up until now, no promising second-line therapeutic options have become available for the management of invasive or metastatic BCa. A novel treatment strategy is urgently needed for the adequate treatment of BCa.

The epithelial-mesenchymal transition (EMT) is a biological process in which epithelial cells lose polarity and acquire a mesenchymal phenotype [4,5] that underlies cellular development and tissue homeostasis in the adult organism [6]. Substantial evidence implicates EMT in cancer progression, such as metastasis, resistance to chemotherapy and acquisition by cancer cells of the stem cell-like phenotype [7,8]. A comprehensive review on the role of EMT in BCa metastasis has revealed that the EMT phenotype is highly correlated with muscle-invasive bladder cancer (MIBC) [9]. That report indicated an inverse correlation between E-cadherin expression and levels of Zeb-1, Zeb-2 and vimentin in BCa cell lines and tumor specimens [9]. Another study has reported the finding that increasing levels of mesenchymal marker expression and higher levels of the critical EMT transcriptional regulator twist are associated with greater disease severity in cases of BCa [10]. Targeting the EMT process may restore the drug sensitivity of BCa cells to conventional intravesical agents.

Autophagy acts as a protective mechanism in cells subjected to starvation or metabolic pressure such as nutrition deprivation, hypoxia, endoplasmic reticulum (ER) stress, and chemotherapeutic agents [11]. During the autophagy process, a double-membraned vesicle forms, encapsulating the cytoplasm and organelles and fusing with the lysosomes that participate in the degradation of the intracellular material [12]. Autophagy plays important roles during the development of numerous diseases, including infections, neurodegenerative and cardiovascular diseases [13]. Importantly, cancer cells induce autophagy to counteract anticancer agents and thus help cells to evade the apoptotic pathway [14]. The molecular mechanism of autophagy regulation involves the mammalian target of rapamycin (mTOR), 5′-AMP-activated protein kinase (AMPK), and extracellular signal-regulated kinase (ERK) [15]. The mTOR kinase that governs the expression of cellular protein is the major inhibitor of autophagy in the presence of growth factors or nutrition-rich conditions, while AMPK controls autophagy in response to low-energy conditions or nutrient deprivation [16]. Autophagy is achieved through multiple processes and many autophagy-related genes either participate in de novo membrane formation, autophagosome formation and fusion of lysosomes to autophagosomes for degradation, or the reuse of engulfed macromolecules [17].

Recently, melatonin has attracted attention for its promising oncostatic activity against different types of tumors [18,19,20]. In a meta-analysis review of clinical data on the use of adjunctive melatonin in the treatment of several different types of cancers, marked clinical benefits with melatonin included improvements in tumor remission rates and overall survival, as well as the relief of side effects associated with the cancer therapies [19]. Melatonin reportedly achieves antimetastatic effects by inhibiting the EMT process [18] via the targeting of nuclear factor kappa B (NF-κB) [21], which contributes to the induction of EMT-promoting transcription factors such as snail, twist, slug and zeb [22,23]. In vivo evidence has shown how melatonin treatment can effectively attenuate lung cancer metastasis in the liver by reversing the EMT phenotype via the Wnt/β-catenin pathway [24]. This study investigated the impact of melatonin on EMT regulation and the subsequent repression of metastasis in an attempt to improve the clinical application potential of melatonin.

## 2. Results

### 2.1. Cell Viability Test in BCa Cells Treated with Melatonin

**Figure 1:**
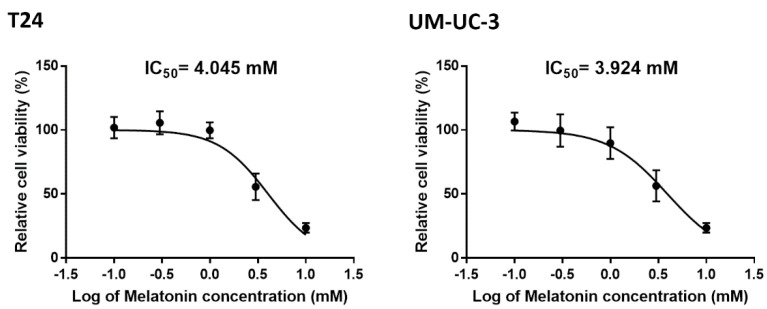
The cell viability of bladder cancer cells after melatonin treatment. T24 and UM−UC−3 bladder cancer cells were incubated with melatonin (0, 0.1, 0.3, 1 or 3 mM) for 24 h, and cell viability was determined by MTT assay. The IC50 were calculated in both cells and provided in the figures.

To assess the effect of melatonin on BCa cells, we first evaluated the viability of BCa cells treated with melatonin. Two BCa cell lines, T24 and UM-UC-3, showed similar cell viability (IC_50_ ≈ 4 mM) in response to melatonin treatment (Figure 1). At concentrations of below 1 mM, melatonin exhibited no significant cytotoxicity in these BCa cell lines.

### 2.2. Melatonin Suppresses BCa Cell Migratory Potential

**Figure 2:**
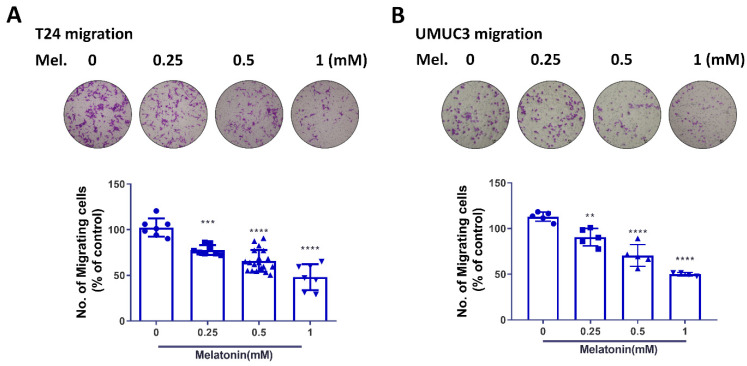

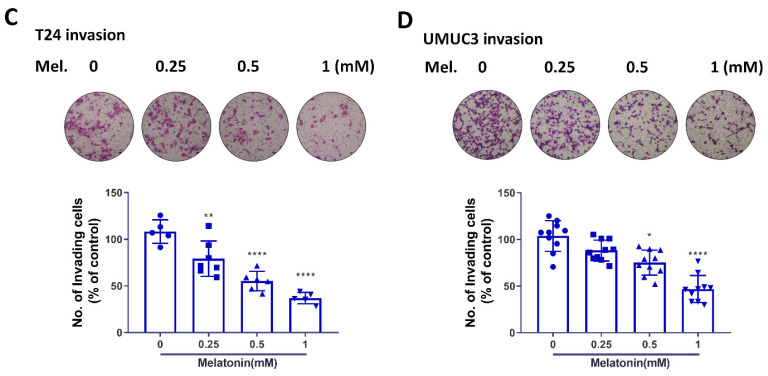
Melatonin suppressed migratory potential of bladder cancer cells. T24 and UM-UC-3 cells, treated with various concentrations of melatonin (0, 0.25, 0.5 and 1 mM), were further subjected to evaluation of cell migratory potential 24 h post-treatment. The in vitro migration (**A**,**B**) and invasion assays (**C**,**D**) were performed using the Transwell to determine cell migratory potential. The results are expressed as the mean ± SEM. * *p* < 0.05, ** *p* < 0.01, *** *p* < 0.001, and **** *p* < 0.0001 compared with the control group.

Based on the cell viability findings after melatonin treatment, the melatonin concentrations from 0.25–1 mM were selected to investigate the effects of melatonin on cell migratory potential without affecting cell viability. The migratory ability, including cell migration and invasion, was dramatically reduced in two high-grade BCa cell lines (T24 and UM-UC-3) in response to melatonin, with the greatest inhibition (≈50%) seen at 1 mM (Figure 2). These results showed that melatonin is able to reduce migratory potential of BCa cells in vitro.

### 2.3. Melatonin Inhibits EMT and Thus Contributes to Antimigratory Effects

**Figure 3:**
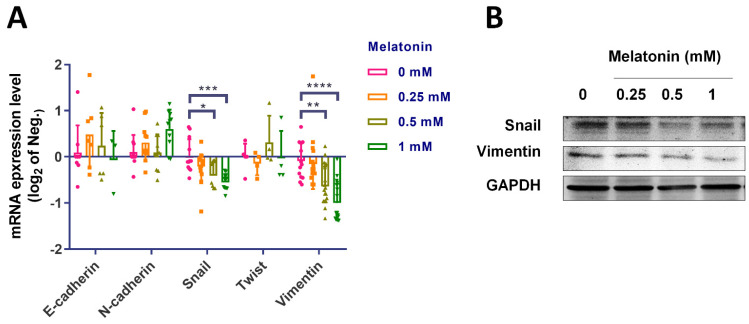
Melatonin inhibited EMT markers in bladder cancer cells. T24 bladder cancer cells, treated with various concentrations of melatonin (0–1 mM) for 24 h, were subjected to analysis of EMT marker expression. The expression levels of E-cadherin, N-cadherin, vimentin, snail and twist were examined by qPCR (**A**) and Western blot (**B**) analysis. GAPDH was used as the internal control. Results are expressed as the mean ± SEM. * *p* < 0.05, ** *p* < 0.01, *** *p* < 0.001, and **** *p* < 0.0001 compared with the control group.

The induction of EMT is highly correlated with the progression of MIBC, and targeting EMT has shown promise as a novel therapeutic strategy. Furthermore, melatonin reportedly exhibits marked antitumoral effects in lung cancer by targeting the EMT process [24]. We therefore examined levels of EMT marker expression in BCa cells after melatonin exposure. Our findings indicate that melatonin may effectively reduce the mRNA levels of mesenchymal markers snail and vimentin (Figure 3A). The protein expressions of snail and vimentin were also decreased as well (Figure 3B). This evidence indicated that melatonin exerted its antimigratory effects by targeting EMT.

### 2.4. Melatonin Reduces Autophagy and Inhibits the Migratory Capacity of BCa Cells

**Figure 4:**
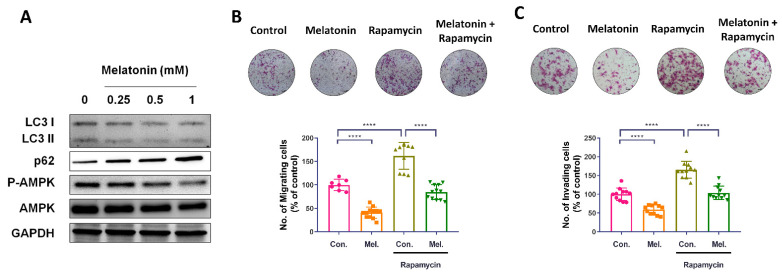
Melatonin-induced inhibition of bladder cell migration was potentiated by autophagy. (**A**) T24 bladder cancer cells, treated with various concentrations of melatonin (0–1 mM) for 24 h, were subjected to analysis of autophagy marker expression. The expression levels of LC3, p62, p-AMPK, and AMPK were determined by Western blot. GAPDH was used as the internal control. (**B**,**C**) T24 and UM-UC-3 cells were pretreated with rapamycin (1 μM) for 30 min before incubation with melatonin (1 mM) for 24 h; cell migration was examined with the Transwell assay. Results are expressed as the mean ± SEM. **** *p* < 0.0001 compared with the control group.

Autophagy is an essential cellular process, during which cells self-digest cytoplasmic components in order to counteract toxic or damaged products [25]. BCa cells exhibit high levels of basal autophagy, which are required for cancer cell growth and survival [26]. Increasing evidence shows that melatonin regulates the autophagic processes involved in physiological and pathological states [27]. We therefore investigated whether melatonin mediates autophagy and is associated with antitumor effects in BCa. We observed reductions in levels of p-AMPK and LC3-II, and p62 accumulation after melatonin treatment (Figure 4A). We also examined whether melatonin-induced inhibition of autophagy is required to reduce the migratory potential of cancer cells. We found that pretreating T24 cells with the autophagy inducer rapamycin prevented the antimigratory effects induced by melatonin incubation (Figure 4B,C).

**Figure 5:**
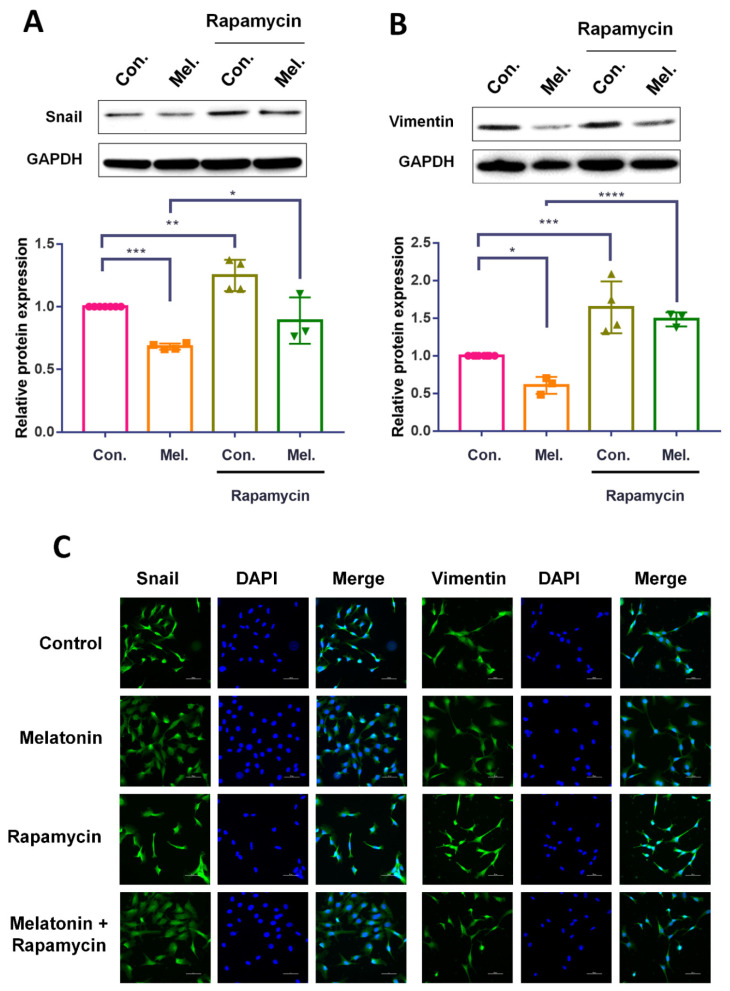
Melatonin-suppressed EMT in bladder cancer was regulated by autophagy. (**A**,**B**) T24 and UM-UC-3 cells were pretreated with rapamycin (1 μM) for 30 min before incubation with melatonin (1 mM) for 24 h. The total cell lysates were collected and subjected to determine snail and vimentin expressions by Western blot. (**C**) The cells seed on chamber slides were treated as described in (**A**), and expression levels of snail and vimentin were monitored by immunofluorescence stain. Results are expressed as the mean ± SEM. * *p* < 0.05, ** *p* < 0.01, *** *p* < 0.001, and **** *p* < 0.0001 compared with the control group.

We also examined whether rapamycin pretreatment impacts upon melatonin-induced alterations in EMT marker expression in BCa cells. Western blot and immunofluorescence data revealed significantly higher levels of snail and vimentin expression in cells pretreated with rapamycin, compared with those treated with melatonin alone (Figure 5A,B). In summary, our finding revealed that melatonin reduced autophagy in BCa cells, contributing to the inhibition of EMT and cell migratory potential.

### 2.5. Intravesical Instillation of Melatonin in an Orthotopic Mouse Model of BCa

**Figure 6:**
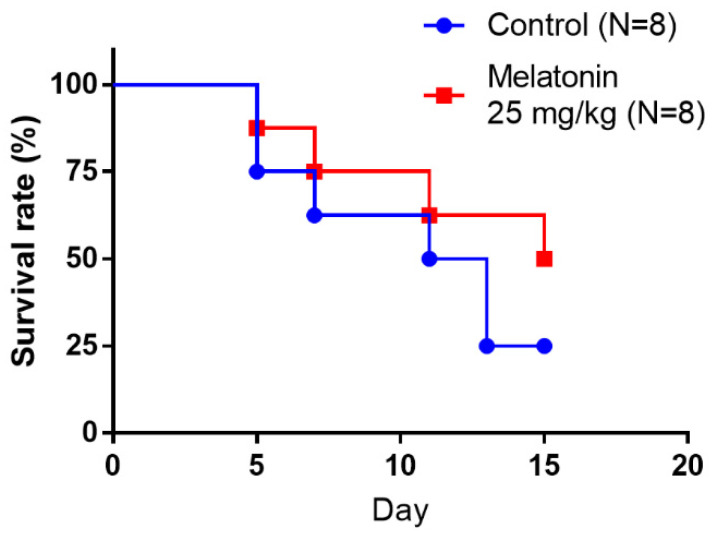
Kaplan–Meier analysis of survival rates of mice according to intravesical instillation of melatonin after UM-UC-3 cells implantation in an orthotopic bladder cancer model. Animals were observed every day for the entire in-life observation period of 20 days. Control (●); Melatonin 25 mg/kg (■).

Finally, we evaluated whether a single dose of melatonin prolongs survival in BCa tumor-bearing mice. A slight survival advantage was observed in the melatonin-treated group (*n* = 8) compared with the vehicle group (*n* = 8). At Day 15, 4 (50%) of the melatonin-treated mice remained alive, whereas only 2 (25%) were alive in the vehicle group (Figure 6). These data suggested that melatonin could prevent BCa progression in vivo.

## 3. Discussion

Increasing evidence indicates that melatonin can inhibit tumor progression by regulating various biological cellular processes such as proliferation, invasion, metastasis and apoptosis [28,29]. Recent research has found that mature epithelial cells can undergo a second round of EMT, by attaining an invasive, motile phenotype that leads to metastasis [5]. In this study, we found that melatonin inhibits the migratory potential of BCa cells and the EMT phenomenon, which may contribute to the antimetastatic effects of melatonin. Furthermore, by suppressing autophagy in BCa cells, melatonin exhibited antimigratory activity. Finally, in vivo results revealed that the intravesical instillation of melatonin increased survival rates of orthotopic xenograft mice. Our results provide new insights for the clinical application of melatonin in BCa treatment.

Our results showed that treatment with 1 mM melatonin exhibited great antimigratory effects in BC cells, which was in agreement with previous reports in several cancer types [30]. Moreover, IC_50_ of melatonin in BCa cells were approximate 4 mM. Previous studies also showed that IC_50_ of melatonin was 4.8 mM in ovarian cancer cells [31], 1.5~2.5 mM in prostate cancer cells [32]. These findings revealed concentration of anti-proliferate activity of melatonin were mostly ranged from 1–5 mM in different cancer cells. In many cell types, concentrations of melatonin less than 1 mM shows no cytotoxicity effect [33,34,35]. Our result also indicated that treatment of 1 mM melatonin did not have cytotoxicity effect on normal cells (Figure S1, Supplementary Material), which was in accordance with previous investigation [36]. In Liu et al. research, the IC50 of cisplatin in BCa cells is about 30 μM [37]. Moreover, in Stravopodis et al. study, the IC50 of doxorubicin in BCa cells is about 20 μM (10 μg/mL) [38]. Our results showed IC50 of melatonin was about 4 mM. Although this concentration is higher than conventional anti-tumor drugs used in BCa treatment (cisplatin and doxorubicin), melatonin still shows promising effects against tumor progression with in vitro and in vivo evidence. The clinical trials also show therapeutic effects of melatonin on BCa patients. In conclusion, this evidence implicates melatonin as a potential drug for use in BCa treatment.

Melatonin shows antimetastatic activity by targeting the cell cycle and apoptosis, by preventing crosstalk between cells, the cell matrix and cell cytoskeletons, and by inhibiting EMT [39]. Several studies have addressed the capacity of melatonin to suppress EMT [18]. In one investigation, melatonin inhibited the invasive capacity of BCa cells by regulating the expression of E-cadherin and β-integrin [40]. Our previous study findings revealed that melatonin inhibits lung cancer metastasis by targeting EMT, which is regulated by twist expression [24]. In contrast, this study demonstrates that melatonin can reduce the levels of mesenchymal markers snail and vimentin, but not twist. The different actions of melatonin may be in the context of cell types. Another study has described how the antiperitoneal dissemination potential of melatonin in vivo appears to inhibit EMT [41]. This evidence emphasizes that melatonin may potentially reduce the primary tumor spread in different types of cancers. Previous reports had discovered the signal pathways involved in cell migratory ability, which is also related to EMT regulation [42,43]. The signal pathway by which melatonin decreases EMT in BCa cells should be addressed in the future.

Autophagy, a stress response cell mechanism that plays an important role in cancer progression, becomes apparent when cancer cells encounter environmental stress, such as hypoxia, energy deprivation or delivery of chemotherapeutic agents [44]. The results from this study indicate that melatonin inhibits autophagy in BCa cells. Melatonin is reported to induce or inhibit autophagy in different conditions. For example, melatonin inhibits carbon tetrachloride-induced autophagy in mice [45]. In TM3 Leydig cell, melatonin can abrogate H_2_O_2_-induced autophagy via the AKT/FOXO1 pathway [46]. During liver ischemia/reperfusion status, melatonin exhibits protect effects by the inhibition of autophagy [47]. This evidence showed that melatonin has a regulatory role in autophagy. Furthermore, the results also show that treatment with the autophagy inducer rapamycin markedly reversed melatonin-induced reductions in cell migratory potential, suggesting a regulatory role of autophagy on antimigratory effects. The correlation between autophagy and EMT has been described in previous reports [48,49]. Based on the dual role of autophagy in cancer progression, the evidence for autophagy in EMT is controversial in the context of the cellular type and on the stimulus employed for activating or inhibiting autophagy. Different compounds and microenvironmental conditions reportedly stimulate EMT change, and induce autophagy in various cell types, whether they are cancerous or non-cancerous. Moreover, evidence indicates that autophagy inhibition impairs the EMT phenomenon in cancer cells [50]. However, some evidence shows that autophagy activation may reverse the EMT phenotype in both healthy and cancerous scenarios, and that several anticancer compounds that induce autophagy also inhibit EMT [50]. Our results in this study support the contention that melatonin inhibits EMT by targeting autophagy in BCa cells. Future research will attempt to elucidate the underlying mechanisms in greater detail.

For muscle-invasive BCa, combinations of methotrexate, vinblastine, doxorubicin and cisplatin are recommended as first-line chemotherapy [51]. However, no definitive recommendations exist for second-line therapy and, despite the established treatment for BCa, recurrence rates remain high. Thus, novel therapeutic options are warranted for BCa treatment. Our study has evaluated the antitumor effects of intravesical administration of melatonin in an orthotopic BCa model. Our results suggest that melatonin slightly prolongs the survival of tumor-bearing mice. According to our findings, it may be that higher melatonin doses are required, or that it may be more beneficial to use melatonin as adjuvant therapy in combination with existing conventional treatment strategies. Our previous study has showed promising response of melatonin treatment in lung cancer in vivo. Melatonin treatment greatly abolished lung cancer metastasis in mice model [24]. In addition, the anti-cancer effect of melatonin has been proved in lung, breast, prostate, colorectal, skin, liver, cervical, and ovarian cancers [52]. Several clinical trials have been conducted to investigate the anti-tumor effect of melatonin in various cancers and showed benefits in cancer treatment [53,54,55]. Few studies have reported the therapeutic effects of melatonin in BCa [56]. However, the in vivo results were limited because of the subcutaneous implantation of BCa tumors. Here, we provided evidence that intravesical instillation of melatonin could prolong the survival of the mice with orthotopic implantation of BCa cells.

## 4. Materials and Methods

### 4.1. Cell Culture

Human BCa cell lines (T24 and UM-UC-3) were obtained from the Bioresource Collection and Research Center (Hsinchu, Taiwan) and maintained at 37 °C under 5% CO_2_ conditions. T24, and UM-UC-3 cells were cultured in RPMI-1640, McCoy’s 5A, or F12K media, respectively. Culture media were supplemented with 10% FBS, 2 mM GlutaMAX-1, 100 U/mL penicillin, and 100 μg/mL streptomycin. Cells were seeded onto 96-well or 6-well plastic plates or onto 10 cm dishes for cell viability assays and for collecting RNA or protein samples. All materials required for cell culture were obtained from Gibco^TM^ (Thermo Fisher Scientific Inc., Waltham, MA, USA).

### 4.2. Cell Viability Assays

Cells were seeded onto 96-well plates before the indicated treatment. Cell viability was assayed using an MTT reagent (Roche Diagnostics, Mannheim, Germany) and determined as a percentage of the control. Samples were protected from light during the assays, and the incubation time for MTT was set at 1 h. Each condition was performed in three replicate wells, and data were obtained from at least three independent experiments.

### 4.3. Migration Assay

The Transwell migration assay was conducted with Transwell plates (Costar, NY, USA; pore size, 8 μm). Briefly, 300 μL of serum-free medium was added into the lower chamber. Meanwhile, 1 × 10^4^ cells were suspended in 100 μL of serum-free medium containing different concentrations of melatonin and indicated inhibitors, then added into the upper chamber. The cells were incubated at 37 °C in 5% CO_2_ for 24 h, before the Transwell inserts were fixed in 3.7% formaldehyde for 15 min. Then, 0.05% crystal violet dissolved in PBS was added to stain the cells for 15 min. The Transwell inserts were washed three times with PBS, and the cells in the upper chamber were removed using cotton swabs. Cells that had migrated to the lower side of the Transwell inserts were observed and counted using a microscope. A minimum of three independent experiments were conducted for each experimental condition.

### 4.4. Immunofluorescence Staining

Cells grown on chamber slides were subjected to immunofluorescence staining. In brief detail, the treated cells were fixed with 4% paraformaldehyde at room temperature. Thirty minutes later, 5% nonfat milk in phosphate-buffered saline (PBS) containing 0.25% Triton X-100 was added to the cells. The cells were then incubated in rabbit anti-snail or anti-vimentin (1:100) and fluorescein isothiocyanate-(FITC)-conjugated goat anti-rabbit secondary antibodies (1:500; Leinco Technology Inc., St. Louis, MO, USA) for 1 h. FITC was detected using a Zeiss fluorescence microscope.

### 4.5. Western Blot Analysis

Cellular lysates were prepared, and proteins were resolved on SDS-PAGE and transferred to an Immobilon polyvinyldifluoride membrane. The blots were blocked with 4% bovine serum albumin (BSA) for 1 h at room temperature and then probed with rabbit anti-human antibodies against candidate apoptotic and autophagic pathway proteins (1:1000) for 1 h at room temperature. After three washes, the blots were incubated with donkey anti-rabbit peroxidase-conjugated secondary antibody (1:1000) for 1 h at room temperature. The blots were visualized with enhanced chemiluminescence using Kodak X-OMAT LS film (Eastman Kodak, Rochester, NY, USA). Quantitative data were obtained using a computing densitometer and ImageQuant software (Molecular Dynamics, Sunnyvale, CA, USA).

### 4.6. Quantitative Real-Time PCR

Quantitative real-time PCR analysis used TaqMan^®^ 1-step PCR Master Mix (Applied Biosystems, Foster City, CA, USA). One hundred nanograms of total cDNA was added for each 25 µL reaction with sequence-specific primers and TaqMan probes. Sequences for all target gene primers and probes were purchased commercially (β-actin served as the internal control; Applied Biosystems, Waltham, MA, USA). Quantitative real-time PCR assays were performed in triplicate on a StepOnePlus™ sequence detection system. Cycling conditions were set at 10 min of polymerase activation at 95 °C, followed by 40 cycles at 95 °C for 15 s and 60 °C for 60 s. The threshold was set above the nontemplate control background and within the linear phase of target gene amplification to calculate the cycle number at which the transcript was detected (denoted as C_T_).

### 4.7. Orthotopic BCa Animal Model

All murine experimental procedures were approved by the Institutional Animal Care and Use Committee of Shin-Kong WHS Hospital and performed according to the hospital’s Guidelines of Animal Experimentation (IACUC No. 109MOST0012). Six-week-old female SCID mice were used for the orthotopic BCa animal model. Confluent UM-UC-3 cells were trypsinized and resuspended in a complete medium/Matrigel (1:1) mix before inoculation. Mice were anesthetized with 3–5% isoflurane in a small chamber and secured by hand. A 24 G Teflon-coated catheter was introduced into the bladder through the urethra. The bladder was treated with trypsin prior to cell instillation. Trypsin (0.125%) with a sterile DMEM base medium (100 μL) was injected by a catheter into the bladder for 30 s to traumatize it. PBS (200 μL) was used to flush the solution in the bladder, before instilling 5 × 10^6^ BCa cells into the bladder. The catheter was removed, and the mice were returned to their cages. Animals were observed every day for the entire in-life observation period of 20 days.

### 4.8. Statistics

All experiments were performed at least three times. Data were analyzed using the Student’s *t* test for statistical significance and expressed as the mean ± standard deviation (SD). *p* < 0.05 was considered to be statistically significant. Survival curves were generated using the Kaplan–Meier method and compared using the log-rank test.

## 5. Conclusions

This study provides novel insights into the application of melatonin as a therapeutic strategy in BCa treatment. Our in vitro and in vivo evidence suggests that melatonin has promising potential in this therapeutic indication. To date, recurrence of bladder cancer after standard treatment is still a clinical issue. Here, our in vivo animal model shows potential application of melatonin on BCa treatment by using intravesical instillation of melatonin. However, the further evaluation of anti-tumor effects of melatonin on orthotopic BCa model should be conducted in the future.

## Data Availability

The data sets used and analysed during the current study are available from the corresponding author on reasonable request.

## Supplementary Materials

The following supporting information can be downloaded at: https://www.mdpi.com/article/10.3390/molecules27248649/s1, Figure S1: The cell viability of normal bladder cells after melatonin treatment.

## Sample Availability

Samples of the compound melatonin is available from the authors.
