# Supplementary material for: Melatonin Inhibits EMT in Bladder Cancer by Targeting Autophagy
Source: Molecules. 2022 Dec 7;27(24):8649. doi: 10.3390/molecules27248649 (PMC9784694; doi:10.3390/molecules27248649)
Supplement: Supplementary file 1 [file molecules-27-08649-s001.zip › molecules-2035679-supplementary.pdf]

Supplementary information

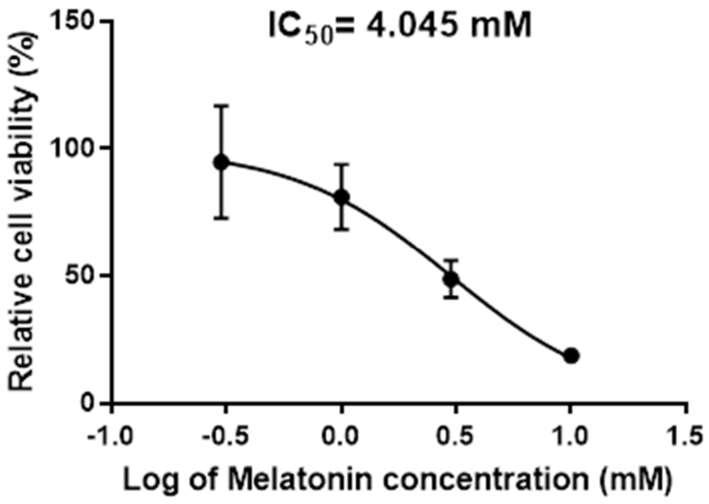

Figure S1. The cell viability of normal bladder cells after melatonin treatment. SV-HUC-1 normal bladder cells were incubated with melatonin (0, 0.1, 0.3, 1 or 3 mM) for 24 h, and cell viability was determined by MTT assay. The IC50 was calculated in both cells and provided in the figures.
